# Cephalometric Study of the Overjet Development in Warmblood Foals

**DOI:** 10.3389/fvets.2019.00431

**Published:** 2019-11-29

**Authors:** Natalia Domanska-Kruppa, Monica Venner, Astrid Bienert-Zeit

**Affiliations:** ^1^Lewitz Stud, Neustadt-Glewe, Germany; ^2^Veterinary Clinic Destedt, Cremlingen, Germany; ^3^Clinic for Horses, University of Veterinary Medicine Hannover, Foundation, Hanover, Germany

**Keywords:** equine cephalometry, overjet development, overjet measurements, overjet regression, class II dental malocclusion, foal

## Abstract

Class II malocclusion is the most frequently occurring congenital malocclusion in horses. Radiographic cephalometric procedures adopted from human dentistry were used to study the development of overjet in a population of 650 Warmblood foals. Thirteen foals were diagnosed with measurable overjet at the beginning of the study. The malocclusion in nine foals resolved spontaneously and four foals without overjet at 2 weeks of age developed the condition during the first year of life. A cephalostat used in human orthodontics to immobilize the patient's head while being radiographed was replaced by a researcher-made head-holding device, whose size was based on the results of a pilot study. Laterolateral digital radiographs of each foal's head (cephalograms) were taken at five time points until the age of 12 months. Thirteen cephalometric points were identified and nine distances were measured on each radiograph. Additionally, the angle between the long axis of the upper and lower incisors was evaluated. Cephalometric measurements proved to be useful to identify foals that showed spontaneous regression of the malocclusion over the study time between 9 and 16 weeks of age.

## Introduction

The prevalence of overjet in the equine population is between 2 and 5% (1–3) and, is thereby, the most frequently occurring congenital malocclusion. The exact prevalence of overjet in humans is difficult to determine because of ethnic differences in the populations sampled and varies from 8.6 to 33.7% (4). Overjet is considered to be congenital, but several reports describe horses acquiring the condition in the first sixth months of life (5–7). Spontaneous regression of overjet in equids was described for the first time in the study of Domanska et al. (3). Affected horses can suffer from diverse clinical consequences, such as injuries of the hard palate behind the maxillary incisors or sharp overgrowths on the mesial aspect of the upper first cheek teeth and on the lingual aspect of the lower last cheek teeth (8). Related pain during food uptake can lead to excessive weight loss as a long-term consequence (9). Riding horses can have bitting problems (10). There is some discussion that class II malocclusion might be hereditary (11), therefore, breeders' associations can exclude affected stallions and mares (12). Several different approaches have been developed for the management and correction of overjet in the horse depending on the severity of the condition. In the case of mild overjet, when the upper incisor teeth protrude labial to the lower incisors, but there is still contact between these teeth, only corrective floating is needed (13). In other cases with mild (<1 cm) incisor overjet in young horses, the malocclusion can be successfully corrected by placing a tension band wire on the upper jaw to retard rostral maxillary and premaxillary growth (5, 14). In foals with an overjet of > 1 cm and developed overbite, in addition to a tension band wire system, an acrylic platform with a metallic plate that is fixed in an inclined position behind the upper incisors is used to create occlusion between the lower incisors and the bite plate. This provides the possibility of correcting the overbite by advanced growing of the mandible (14–17). Osteodistraction is a recommended method for the treatment of the suspected mandibular brachygnathia (18). Verwilghen et al. (15) described mandibular elongation and correction of brachygnathia achieved by osteotomy of the mandible and placing a bite plate on the upper incisors, and Lischer (19) described a modified lengthening osteotomy for such cases. Successful surgical management using temporary application of premaxillary tension band devices is possible until the age of 12 months (5). The best results were achieved when the foals were younger than 6 months (5–7, 20). Other authors recommend that treatment begins as soon as possible, because increased age at the beginning of the therapy influences the correction rates negatively (21). Because time is a limiting factor, it is essential for the breeders to know when they should decide on surgery and how overjet progresses in untreated foals.

The crucial diagnostic tool used in human orthodontics to investigate overjet behavior and to plan a therapy is cephalometric analysis. The cephalogram used for this procedure is a two-dimensional, lateral x-ray image of the skull. It is helpful to evaluate dentofacial proportions before the treatment and predict future dentofacial changes due to orthodontic correction (22). Bony landmarks (so-called cephalometric landmarks) are located on the radiograph to diagnose the patient's deviation from normative values. Linear distances and angles among these landmarks are evaluated to describe craniofacial growth discrepancies (23). Several cephalometric studies have been conducted on the heads of different species, such as monkeys (24, 25), dogs (26), rats (27, 28), pigs (29, 30), miniature pigs (31), rabbits (32, 33), cats (34), and sheep (35).

The aim of this study was to develop a radiological cephalometric method for evaluating longitudinal changes in skull bone dimensions in Warmblood foals. Furthermore, whether radiological examinations could be useful for predicting overjet resolution in Warmblood foals was addressed. An attempt was made to determine the age at which overjet develops or resolves in growing horses. Among all synonyms of mandibular distoclusion (class II malocclusion, brachygnathism, mandibular brachygnathism, overjet) the authors decided to use the term “overjet” throughout the manuscript to emphasize that the all the measurements concerned the horizontal projection of maxillary incisors beyond the mandibular incisors and none of the foals had an overbite.

## Materials and Methods

Pilot research with 20 foals was carried out to determine the optimal head and neck position for head radiography in a neutral position. A custom-made head-holding device was developed to reproduce a so-called *standard examination position* while being radiographed (3). All newborn foals from one foaling season at a Warmblood stud in Germany were investigated in this study. They were examined for any incongruity in the relationship between maxilla and mandible at 2 weeks of age, with the incisors viewed from the side. If any protrusion of the upper incisors in relation to lower incisors was seen, the foals were included in the *overjet group* of the study. At the same time, one healthy, randomly selected foal born on the same day was selected for the *control group*. All foals selected for the study were radiographed at five time points until they were 12 months old. At every time point, the distance (in mm) between the occlusolabial edge of the central upper and lower incisors was measured using a tire tread depth gauge (3). The first radiological investigation was performed on the foals when they were three to 8 weeks old. Subsequently, x-rays and overjet measurements were performed three times until weaning at 6 months of age. The last radiographs and measurements were taken at 11 or 12 months of age.

As some foals classified with regular incisor alignment at 2 weeks of age developed overjet later and some foals born with incisor misalignment recovered during the study period, it was necessary to divide those foals into two additional groups to describe this overjet development or resolution. One group consisted of control foals which developed overjet during the study: These were called the *positive control group*. Another group consisted of foals with overjet at 2 weeks of age which showed spontaneous regression of the malocclusion over time: These were called the *negative overjet group*.

All foals which showed incisor misalignment at 2 weeks of age were retrospectively assigned to the *overjet* or the *negative overjet group* to compare the parameters between the individuals in which overjet persists and those in which the overjet resolved by 16 months of age. The group of foals that showed overjet regression became smaller over time because the regression of overjet could occur at any time between the first and the fifth measurement point. The foals affected by overjet whose disorder resolved by the third measurement, for example, were classified among the *negative overjet* group during the first and the second measurements, but after the malocclusion corrected spontaneously, they were no longer included in this group during future measurements. Analogous findings apply to the situation among the *control* and *positive control group* (3).

**Figure 1:**
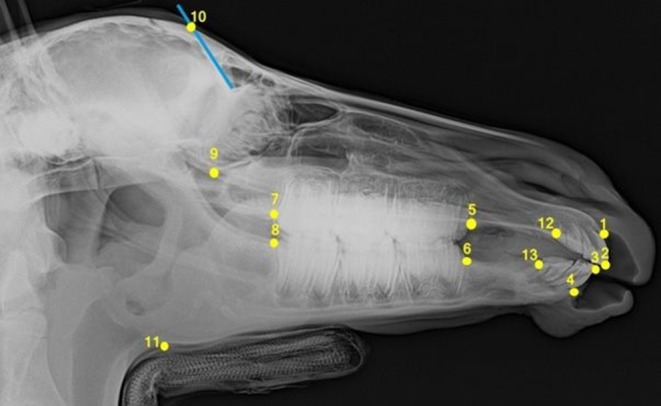
Laterolateral radiograph of the foal's head with cephalometric landmarks described in detail in Table 1.

**Figure 2:**
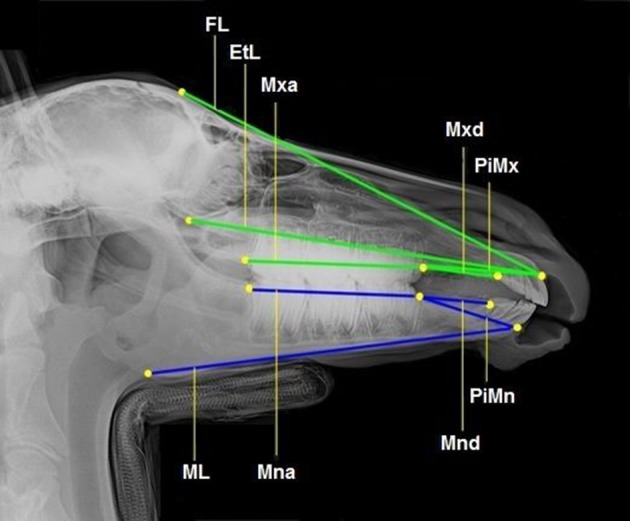
Laterolateral radiograph of the foal's head with cephalometric lines described in detail in Table 2.

All foals were intravenously sedated with 0.8 mg/kg xylazine (Xylazin 2%®, Riemser Arzneimittel AG, Greifswald-Insel Riems, Germany) for radiographic examination. After bringing the foal into the *standard examination position* with the head and neck fixed to the head support (3), laterolateral radiographs were obtained. All the radiographs were taken in the same projection with the x-ray source situated 115 cm lateral to the foal and the x-ray plate immediately lateral to foal's head. Each radiograph was analyzed according to the same procedure. Thirteen landmarks were determined on the lateral radiographs of the foal's head (Figure 1). Nine cephalometric lines were measured (Figure 2).

**Table 1:** Description of the cephalometric points.

	**Cephalometric point**	**Abbr**.	**Description**
1	Incisive bone cusp	IBC	The most rostral point of the incisive bone between the first maxillary incisors
2	Tip point A	TA	Incisal tip of the most labially placed maxillary first incisor
3	Tip point B	TB	Incisal tip of the most labially placed mandibular first incisor
4	Mandibular alveolar process cusp	MAPC	The most rostral point on the mandibular alveolar process between the first mandibular incisors
5	Premolar superior	Ps	The point formed by the intersection of the mesial margin of the second premolar of the upper jaw with the interdental space of the maxilla
6	Premolar inferior	Pi	The point formed by the intersection of the mesial margin of the second premolar of the lower jaw with the interdental space of the mandible
7	Molar superior	Ms	The point formed by the intersection of the distal margin of the last occluded molar of the upper jaw with the margin of the maxilla
8	Molar inferior	Mi	The point formed by the intersection of the distal margin of the last occluded molar of the lower jaw with the margin of the mandible
9	Inferior ethmoturbinate	Eti	The lowest point of the inferior margin of the ethmoid turbinate within the orbit
10	Frontal sinus	FS	The most caudal point of the frontal sinus that can be easily identified on the radiograph
11	Mandibular angle	Man	The most caudal located inferior point on the angle of the mandible (approx. in the middle of the convexity of the mandible angle)
12	Incisor superior	SuI2	The point formed by the intersection of the distal margin of the last erupted incisor of the upper jaw with the interdental space of the maxilla
13	Incisor inferior	InI2	The point formed by the intersection of the distal margin of the last erupted incisor of the lower jaw with the interdental space of the mandible

**Table 2:** Cephalometric lines description.

**Cephalometric line**	**Abbr**.	**Line connecting…**
Facial line	FL	… the frontal sinus (FS) and incisive bone cusp (IBC)
Ethmoid line	EtL	… the inferior ethmoturbinate (ETi) and incisive bone cusp (IBC)
Maxillary cheek teeth length	Mxa	… the molar superior (Ms) and premolar superior (Ps)
Pars incisiva maxillae	PiMx	… the premolar superior (Ps) and incisive bone cusp (IBC)
Maxillary diastema	Mxd	… the premolar superior (Ps) and incisor superior (SuI2)
Mandibular cheek teeth length	Mna	… the premolar (Pi) and molar inferior (Mi)
Mandibular diastema	Mnd	… the premolar inferior (Pi) and incisor inferior (InI2)
Pars incisiva mandibulae	PiMn	… the premolar inferior (Pi) and mandibular alveolar process cusp (MAPC)
Mandibular length	ML	… the alveolar process cusp (MAPC) and mandibular angle (Man)

**Figure 3:**
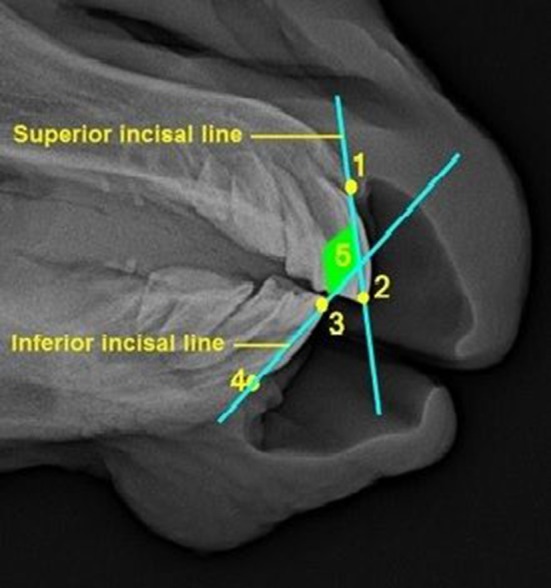
Rostral part of a laterolateral radiograph of the foal's head with incisal lines and interincisal angle (described in detail in Table 3).

An interincisal angle was measured to investigate the relative position of the upper and lower incisors to each other. This angle is created by the intersection of the *superior incisal line* and *inferior incisal line* (Figure 3 and Table 3). Nineteen rations were determined out of nine cephalometric lines drawn to investigate overjet evolution and regression in 13 foals within the first 12 months of life.

**Table 3:** Description of the lines forming the interincisal angle.

	**Cephalometric line/angle**	**Description**
1–2	Superior incisal line	The line connecting the incisive bone cusp (IBC) and tip point A (TA)
3–4	Inferior incisor line	The line connecting tip point B (TB) and the mandibular alveolar process cusp (MAPC)
5	Interincisal angle	The intersection of the superior incisal and inferior incisal line

Each radiograph was evaluated three times by the same observer (Domanska-Kruppa) during three sessions, to control the accuracy of the measurements. There were 2-week intervals between the evaluations. All cephalometric points and lines were again drawn and measured during every new session and the mean of all three measurements was used for the study. The mean absolute error was estimated to compare the accuracy of linear measurements.

All the procedures, such as the foals' registration, image preview, measurement of distances and angles, were carried out using image processing software *dicomPACS*®*vet* (Oehm and Rehbein GmbH, Rostock, Germany). Measuring data were collected on a spread sheet (Excel® 2010, Microsoft® Corporation Redmond, Washington, USA).

The values measured were used to create 19 measurement ratios for comparison of foals' heads to describe overjet development. The following ratios were compared: EtL/ML, EtL/PiMn, EtL/Mnd, FL/PiMn, FL/ML, ML/Mnd, Mna/ML, Mxa/Mna, Mxa/EtL, Mxd/Mnd, Mxa/PiMx, PiMn/ML, PiMn/Mnd, PiMn/Mxd, PiMx/FL, PiMx/EtL, PiMx/PiMn, PiMx/Mxd, and PiMx/Mnd. Furthermore, the distance between the upper incisor teeth protruding labial and the lower incisors was measured using a tire tread depth gauge.

The purpose of the statistical comparison of the aforementioned ratios was to find out which ones allow the differentiation between: (A) The *overjet group* (foals born with overjet in which this malocclusion persists) and *negative overjet group* (foals that showed spontaneous regression of overjet); (B) the *control group* (foals with regular incisor alignment during the whole study) and *positive control group* (foals with normal occlusion at 2 weeks of age that developed overjet during the study).

A student's *t* test was performed to compare those 19 ratios between the *overjet group* and *negative overjet group* and *control group* and *positive control group*. The means and standard deviations of the parameters measured were defined. *P* values < 0.05 were considered statistically significant. The diagrams were based on mean values and 95% CIs. Calculations were performed with a statistical software package, R V.3.0.2 [R Development Core Team, (36)] and Statistical Analysis System (SAS Institute).

## Results

A pilot study of the foal's body position helped to describe a *standard examination position* in order to standardize the position of the foals while being radiographed (3).

A total of 650 foals were born in a Warmblood stud in Germany in 2011. Thirteen foals (2%) were identified with overjet 2 weeks postpartum. The overjet measured in the foals of this group ranged from 4 to 8 mm at the beginning of the study. None of the foals had an overbite. Nine of 13 foals (69%) showed spontaneous resolution of incisor misalignment between 9 and 29 weeks of age. Four of the 13 control foals (31%) born with physiological occlusion developed an overjet (2–8 mm) between 9 and 22 weeks of age.

Five of the ratios that were tested proved to be helpful in analyzing the progression or resolution of the overjet in the foals examined (Table 4).

**Table 4:** Usefulness of ratios in assessment of overjet development.

**Ratio investigated**		**Usefulness for investigation**		**Age (in weeks) at significant measuring point**
		**YES**	**NO**	
EtL/ML	Ethmoidal line/Mandibular length	x		9–16
EtL/PiMn	Ethmoidal Line/Pars incisiva mandibulae		x	
EtL/Mnd	Ethmoidal Line/Mandibular diastema		x	
FL/PiMn	Facial line/Pars incisiva mandibulae		x	
FL/ML	Facial line/Mandibular length		x	
ML/Mnd	Mandibular length/Mandibular diastema		x	
Mna/ML	Mandibular cheek teeth length/Mandibular length	x		9-16
Mxa/Mna	Maxillary cheek teeth length/Mandibular arcade length		x	
Mxa/EtL	Maxillary cheek teeth length/Ethmoid line		x	
Mxd/Mnd	Maxillary diastema/Mandibular diastema	x		9-16
Mxa/PiMx	Maxillary cheek teeth length/Pars incisiva maxillae		x	
PiMn/ML	Pars incisiva mandibulae/Mandibular length		x	
PiMn/Mnd	Pars incisiva mandibulae/Mandibular diastema	x		9-16
PiMn/Mxd	Pars incisiva mandibulae/Maxillary diastema		x	
PiMx/FL	Pars incisiva maxillae/Facial line		x	
PiMx/EtL	Pars incisiva maxillae/Ethmoidal Line		x	
PiMx/PiMn	Pars incisiva maxillae/Pars incisiva mandibulae	x		3-8
PiMx/Mxd	Pars incisiva maxillae/Maxillary diastema		x	
PiMx/Mnd	Pars incisiva maxillae/Mandibular diastema		x	

Three ratios (Etl/ML, Mxd/Mnd and PiMn/Mnd) were shown to be useful to differentiate foals born with overjet in which the malocclusion persisted (*overjet group*) and foals in which the overjet resolved during the study period (*negative overjet group*). The PiMn/Mnd and Mxd/Mnd ratios were significantly lower between 9 and 16 weeks of age in the *negative overjet group* (Tables 5, 6). The EtL/ML ratio was higher in the *negative overjet group* at the same age (Table 7).

**Table 5:** Pars incisiva mandibulae to mandibular diastema ratio (PiMn/Mnd).

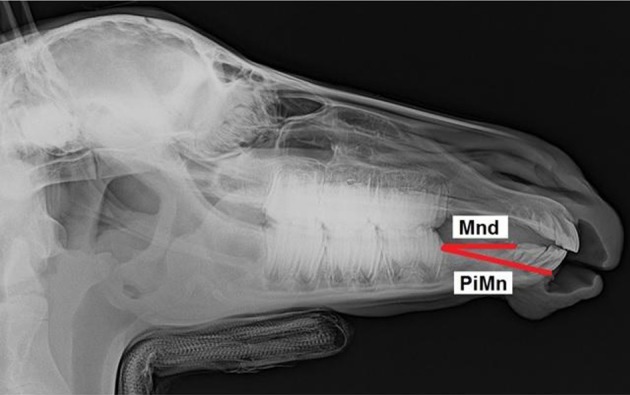					
**Age (weeks)**	**Overjet**		**Negative overjet**		***p***
	***m***	***sd***	***m***	***sd***	
3–8	1.54	0.07	1.45	0.14	0.243
9–16	1.38	0.06	1.27	0.07	0.029
17–22	1.23	0.05	1.21	0.07	0.589
23–29	1.16	0.09	1.16	0.08	0.992

*m, mean; sd, standard deviation*.

**Table 6:** Maxillary diastema to mandibular diastema ratio (Mxd/Mnd).

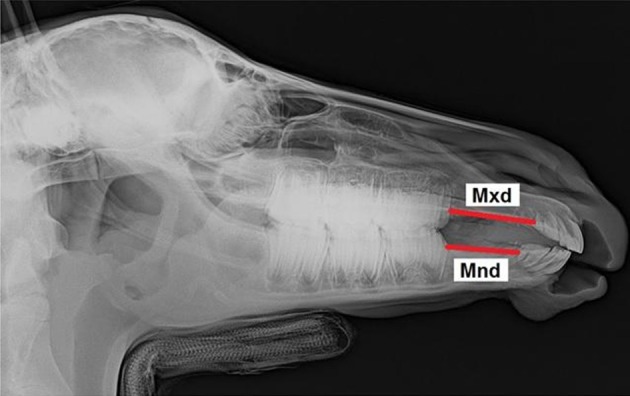					
**Age (weeks)**	**Over jet**		**Negative overjet**		***p***
	***m***	***sd***	***m***	***sd***	
3–8	1.18	0.01	1.15	0.08	0.505
9–16	1.17	0.02	1.09	0.06	0.038
17–22	1.1	0.03	1.06	0.05	0.158
23–29	1.12	0.09	1.07	0.09	0.45

**Table 7:** Ethmoidal line to mandibular length ratio (EtL/ML).

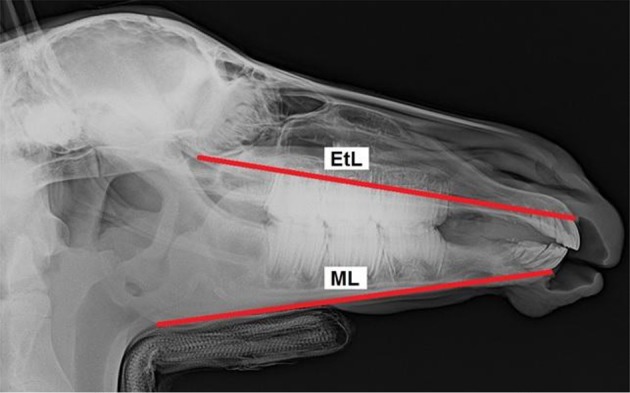					
**Age (weeks)**	**Overjet**		**Negative overjet**		***p***
	***m***	***sd***	***m***	***sd***	
3–8	0.94	0.04	0.97	0.04	0.232
9–16	0.96	0.02	0.99	0.02	0.008
17–22	0.97	0.04	1	0.03	0.171
23–29	0.98	0.02	1	0.04	0.258

Two ratios (PiMx/PiMn and Mna/ML) were significantly higher when comparing the *positive control group*, which developed overjet during the study, with the *control group*. The PiMx/PiMn ratio was higher between 3 and 8 weeks of age and the Mna/ML ratio between 9 and 16 weeks of age (Tables 8, 9).

**Table 8:** Pars incisiva maxillae to pars incisiva mandibulae ratio (PiMx/PiMn).

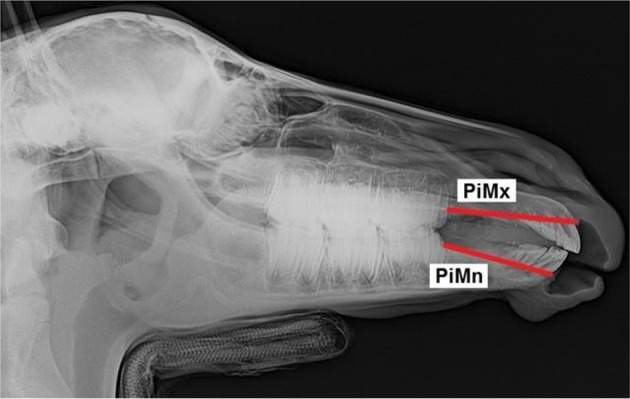					
**Age (weeks)**	**Control**		**Positive control**		**p**
	**m**	**sd**	**m**	**sd**	
3–8	1.17	0.04	1.22	0.02	0.045
9–16	1.17	0.08	1.21	0.01	0.48
17–22	1.19	0.05	1.14	–	1.413
23–29	1.17	0.03	–	–	–

**Table 9:** Mandibular cheek teeth length to mandibular length (Mna/ML) ratio.

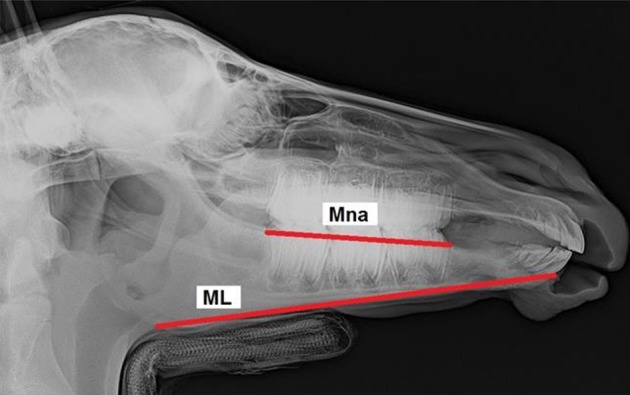					
**Age (weeks)**	**Control**		**Positive control**		***p***
	***m***	***sd***	***m***	***sd***	
3–8	0.48	0.02	0.56	0.012	0.082
9–16	0.43	0.01	0.47	0.01	0.001
17–22	0.43	0.06	0.41	–	0.75
23–29	0.5	0.02	–	–	–

The measurements of the interincisal angle did not show any significant differences between the groups.

The most accurate of the nine linear measurements were the FL—facial (mean absolute error 0.48%) and EtL—ethmoidal (mean absolute error 0.66%) cephalometric lines and the most frequent errors by repeated measurements appeared at the Mxd—maxillary diastema (mean absolute error 2.42%) and Mnd—mandibular diastema (mean absolute error 2.2%) cephalometric lines.

## Discussion

Cephalometry was first introduced in human dentistry by Broadbent (37) as a tool to objectively study craniofacial growth and development. A head-positioning device called a *cephalostat (cephalus* meaning head and *-stat* meaning fixed position*)* is used to place the patient's head in a position which can be reproduced later, whenever required (38). A lateral head radiograph, called a *cephalogram*, is performed to study the correlation between bones, teeth, and soft tissues in human orthodontics (39). A standardized, reproducible head position is essential for obtaining accurate cephalograms (40). Unfortunately, head-holding equipment used in human cephalometric research is not appropriate for horses. In practice, considerations about cephalometry in veterinary medicine led McKeown (41) to highlight the need to build equipment especially designed for the species studied. Therefore, pilot investigations were performed to determine the optimal head-holding device necessary to make cephalometric measurements (3). Some difficulties associated with the head-holding device occurred. Firstly, sedation of the foal leads to muscle relaxation which caused unnatural head and neck positions. This was compensated for by using a head-holding device that standardized the head position in all foals in the study.

Measuring the incisor misalignment using a tire tread depth gauge proved to be an easy and precise method of determining incisor overjet (3). Based on those measurements, it was easy to monitor the overjet development and regression. Some researchers found that overjet can develop between the first and sixth month of life in some foals born with normal occlusion (5–7, 42). This was also confirmed in the current population (3). By contrast, spontaneous regression of overjet within the first 12 months of life has been reported for the first time (3). This phenomenon has also been described in some breeds of dogs (43).

The accuracy of every cephalometric measurement depends on exact landmark identification on the lateral radiograph of the foal's head, which is essential for proper measurement of distances or angles. Errors from landmark identification are the most significant factors in cephalometric analysis in humans and have the greatest meaning for short distances that are measured: The shorter the measuring line is, the greater the inaccuracy that appears (44). The two main causes of measurement inaccuracy are the difficulty in the interpretation of the landmark definitions and the inability of examiners to repeat the landmark position exactly. Landmarks are not always easy to distinguish. The correct interpretation needs a long training time (23). Similar methods to those in the studies of Godfrey and Lertnimulchai (44) were used to ensure the accuracy of the measurements in the current study. Linear measurements on each cephalogram were repeated three times by the same observer during three different times. There were 2-week intervals between each session. The results show that short lines (Mxd and Mnd) showed less accuracy, and long lines showed (EtL and FL) improved accuracy. These findings are in accordance with the findings of researchers in human orthodontics (44).

Three ratios—PiMn/Mnd, Etl/ML, and Mxd/Mnd—proved to be useful to differentiate between foals born with overjet in which the malocclusion persisted *(overjet group)* and affected foals that spontaneously corrected the malocclusion *(negative overjet group)* between 9 and 16 weeks of age. Therefore, those ratios could be used to predict whether foals affected by overjet could recover without surgical intervention. According to many authors, the best results of surgical treatment can be achieved when the foals are younger than 6 months (5–7, 45). Direct measurements of a foal's head described by Domanska et al. (3) allow one to recognize the foals that will not correct overjet spontaneously before 17–22 weeks of age. This is surprisingly late and differs from Easley et al. (21) conclusion that an animal's age influences the degree of possible overjet correction negatively. The cephalometric method presented here allows the actual investigation of the progression of incisor malocclusion early enough to decide whether surgical treatment is needed. However, the small number of the foals in the study makes it questionable how certain the statements are.

Furthermore, the study showed that two ratios (PiMx/PiMn and Mna/ML) were useful to determine whether a foal born with physiological incisor alignment would subsequently develop overjet. This allows the detection of descendants of stallions and mares affected with overjet at an early stage in their lives. Therefore, the foals should be examined for malocclusion between 3 and 8 weeks of age for PiMx/PiMn ratio and between 9 and 16 weeks of age for Mna/ML ratio. The parameter “facial crest line” was repeated in several ratios investigated in the previous study with direct head measurements (3). All of them were useful to differentiate foals born with a normal occlusion where normal incisor alignment persisted until the end of the study (*control group*) and foals with normal occlusion at 2 weeks of age that developed overjet subsequently (*positive control group*). The parameter “facial crest line” runs through the incisive bone and the maxilla. Based on this fact, it was deduced that the maxilla or premaxilla has undergone some elongation. Our initial expectation was that cephalometrical analysis would confirm the previous findings. However, the present study failed to demonstrate that only one specific anatomical area is responsible for overjet development. All the useful ratios (EtL, Mxd, ML, Mna, PiMn, Mnd, PiMx) contained different components of which some were repeated twice (PiMn, Mnd, ML), however, most of them are located outside the premaxilla or the maxilla. Therefore, the best analysis of skull growth can be achieved by using radiopaque markers during an x-ray cephalometric investigation. Such a method involves the use of metallic implants to study bone growth. It is widely used in a variety of experimental animals (primates particularly) (41). Unfortunately, placing metal pins below the periosteum with a specially designed gun is invasive which makes it ethically undesirable in a foal. Another disadvantage is the fact, that erupting tooth germs can cause implants displacement. It would be necessary to find areas of the maxilla and mandible that are free from tooth erupting changes (41).

In humans, the ability to determine the position of a patient's incisors using cephalometric radiographs enhances the orthodontic diagnosis and treatment planning (22). Angular measurements have proved to be suitable for this purpose, and the interincisal angle is the most frequently used. The interincisal angle is the angle between the long axis of the most prominent (anteriorly positioned) maxillary and mandibular first incisors (46). We tried, for the first time, to use the interincisal angle measurements in equine cephalometry. In other species, for example, sheep, the researchers also transferred interincisal angles which were needed in their investigation (35). However, the findings of the present study demonstrate that there are no statistically significant differences regarding the interincisal angle between the groups of foals investigated. It confirms that overjet in horses is not dependent on the inclination of the upper and lower incisors to each other, like in humans (46), but on the lengthening or shortening of the supporting bones. A potential method to investigate craniofaciodental growth could be a longitudinal cephalometrical study with radiopaque markers (44, 47–49). Cephalometric points and lines created in the present study could be useful for this purpose in future investigations.

The main limitations of the study were: small number of affected foals and low severity of overjet, with only 8 mm in the worst case. Therefore, the statements about spontaneous regression of overjet should be considered with caution. The accuracy of cephalometric measurements depends on the experience of the examiner because repeatability of the identification of cephalometric points and lines is crucial for exact measurements. In the current study, the mean absolute error of linear measurements was estimated only for one examiner. It is possible in future investigations to repeat the tracing procedure of each radiograph by at least two examiners to allow the calculation of the intra- and inter-observer variability. Another limitation of the study was the unequal age of the foals within the same measuring time point, for example, between 9 and 16 weeks for the second measuring time point; however, due to the nature of work in the stud, it was not possible to carry out the measurements of all foals at exactly the same age. The late age for the first radiological investigation, between three and 8 weeks, was also caused by working habits in the stud. In order to be able to provide the clinician with some practical guidelines on how to predict wheather correction will occur spontaneously or not we should redo the study on a larger group of foals. An appropriate population could be Quarter Horse foals according to higher prevalence of overjet lesions in these foals (50). Another practical consideration would be to determine cut-off values of the relevant ratios that should be calculated at a proposed specific age. Based on those cut-off values the clinicians could determine the likelihood of spontaneous correction or persistence of overjet.

## Conclusion

The present study is the first radiographic age-determinant cephalometric study performed in horses. The attempt to adopt human cephalometric methods for evaluating overjet development in foals under 12 months proved to be effective, although additional studies of growth patterns with radiopaque markers placed in skull bones could be helpful to understand which bones exactly are involved in overjet development. Our findings about the possibility of recognizing foals born with overjet in which the malocclusion resolves spontaneously at the early age of 9–16 weeks allows one to make more informed decisions about the need for surgical intervention; it is much earlier than in the case of direct head measurement [around the 22nd week, (3)]. Unfortunately, none of the components of the ratios investigated repeated often enough to suggest which bones are directly involved in overjet development.

## Data Availability Statement

The datasets generated for this study are available on request to the corresponding author.

## Ethics Statement

The animal study was reviewed and approved by Niedersächsisches Landesamt für Verbraucherschutz und Lebensmittelsicherheit (LAVES), Dez. 33 Postfach 3949 26029 Oldenburg. Written informed consent was obtained from the owners for the participation of their animals in this study.

## Author Contributions

AB-Z and ND-K designed the study. AB-Z and MV were involved in planning and supervised the work and aided in interpreting the results and worked on the manuscript. ND-K carried out the experiments and performed the measurements and wrote the manuscript with input from all authors. All authors meet the criteria for authorship, participated in the review and the editing of the manuscript, and discussed the results and contributed to the final manuscript.

### Conflict of Interest

MV was employed by the company Altano GmbH. The remaining authors declare that the research was conducted in the absence of any commercial or financial relationships that could be construed as a potential conflict of interest.
